# EGF stimulates human trophoblast cell invasion by downregulating ID3-mediated KISS1 expression

**DOI:** 10.1186/s12964-021-00783-2

**Published:** 2021-10-07

**Authors:** Lanlan Fang, Yibo Gao, Zhen Wang, Yuxi Li, Yang Yan, Ze Wu, Jung-Chien Cheng, Ying-Pu Sun

**Affiliations:** Center for Reproductive Medicine, Henan Key Laboratory of Reproduction and Genetics, The First Affiliated Hospital of Zhengzhou University, 40, Daxue Road, Zhengzhou, 450052 Henan China

**Keywords:** EGF, KISS1, ID3, Trophoblast invasion, Preeclampsia

## Abstract

**Background:**

During pregnancy, trophoblast cell invasion needs to be finely controlled. Aberrant trophoblast cell invasion is associated with placental diseases. Epidermal growth factor (EGF) and its receptor, EGFR, are expressed in trophoblast cells. Although the pro-invasive effect of EGF on trophoblast cells has been reported, the underlying mechanism remains largely unknown.

**Results:**

In the present study, we conducted an RNA sequencing (RNA-seq) to HTR-8/SVneo human trophoblast cells in response to EGF and identified KISS1 as a target gene of EGF. The human KISS1 gene encodes kisspeptin, also known as metastin, which can suppress tumor metastasis. Our results showed that EGF treatment downregulated KISS1 expression and secretion by activating the EGFR-mediated PI3K/AKT signaling pathway. In addition, the expression of inhibitor of DNA-binding protein 3 (ID3) was downregulated by EGF and that was required for the EGF-suppressed KISS1 expression. Functionally, transwell invasion assays demonstrated that EGF stimulated human trophoblast cell invasion by downregulating KISS1 expression. Preeclampsia (PE) is a placental disease characterized by insufficient trophoblast cell invasion. Our clinical results revealed that serum levels of EGF were downregulated while serum and placental levels of KISS1 were upregulated in PE patients.

**Conclusions:**

This study demonstrates that downregulation of EGF can lead to poor trophoblast cell invasion by increasing KISS1 expression which subsequently contributes to the pathogenesis of PE.

**Video Abstract**

**Supplementary Information:**

The online version contains supplementary material available at 10.1186/s12964-021-00783-2.

## Background

Epidermal growth factor receptor (EGFR) belongs to the family of receptor tyrosine kinase that regulates various biological functions such as cell proliferation, migration, invasion, and differentiation. EGF is a polypeptide growth factor that binds specifically to EGFR [1]. At 4–5 weeks of gestation age, EGF and EGFR are expressed in the cytotrophoblast cells of the placenta where they stimulate cell proliferation. At 6–12 weeks of gestation age, the expressions of EGF and EGFR are detected in the syncytiotrophoblast cells of the placenta where they stimulate the productions of important placental hormones such as human chorionic gonadotropin and human placental lactogen production [2]. Reduced expression of EGFR is observed in many pregnancy-related complications including preeclampsia (PE), intrauterine growth restriction (IUGR), and recurrent spontaneous abortion [3]. In contrast, EGFR is overexpressed in hydatidiform mole and choriocarcinoma [4, 5]. Collectively, these studies indicate that EGF/EGFR system plays important role in the regulation of trophoblast function as well as in placenta health and disease.

PE is a leading cause of maternal and perinatal mortality and morbidity that complicates 2–8% of pregnancies [6]. The insufficient trophoblast invasion-induced inadequate remodeling of the uterine vasculature has been considered as a major factor that contributes to the pathogenesis of PE [7]. In addition to EGFR, the plasma levels of EGF in PE patients are significantly downregulated when compared to those in control women [8]. EGF is known to stimulate the invasiveness of human trophoblast cells [9]. EGF stimulates human trophoblast cell invasion by inducing the expression of matrix metalloproteinase-2 (MMP-2) and MMP-9 [10, 11]. In addition, upregulations in the expression of urokinase plasminogen activator (uPA), plasminogen activator inhibitor-1 (PAI-1), and tissue inhibitor of metalloproteinases-1 (TIMP-1) are also involved in EGF-stimulated trophoblast cell invasion [12, 13]. Although the underlying mechanisms that mediate the pro-invasive effect of EGF have been examined, most studies mainly focus on the regulation of extracellular matrix (ECM) degradation and remodeling. Therefore, in the present study, we applied transcriptome analysis to explore the underlying mechanisms that mediate the EGF-stimulated human trophoblast cell invasion.

## Materials and methods

### Cell culture and reagents

The HTR-8/SVneo cell line was obtained from American Type Culture Collection through an official distributor in China (Beijing Zhongyuan Limited). HTR-8/SVneo is an SV40 large T antigen immortalized first-trimester short-lived human trophoblast cell line [14]. HTR-8/SVneo cells were cultured in a humidified atmosphere containing 5% CO_2_ and 95% air at 37 °C in Dulbecco’s modified Eagle’s medium/nutrient mixture F-12 Ham medium (DMEM/F-12; Gibco) supplemented with 10% charcoal/dextran-treated FBS (HyClone), 100 U/mL penicillin, and 100 μg/mL streptomycin sulfate (Boster). EGF, AG1478, AG825, and LY294002 were obtained from Sigma. U0126 was obtained from Cayman. Kisspeptin-10 was obtained from Tocris.

### RNA sequencing and analysis

HTR-8/SVneo cells were treated with vehicle control (DMSO) and 100 ng/mL EGF for 24 h. After treatments, total RNA from each sample was extracted using TRIzol (Thermo Fisher Scientific) according to the manufacturer’s instructions. RNA purity was assessed using a NanoPhotometer spectrophotometer (IMPLEN), and RNA integrity was assessed using the RNA Nano 6000 Assay Kit of the Bioanalyzer 2100 system (Agilent Technologies). Sequencing libraries were generated using the NEBNext® Ultra™ II RNA Library Prep Kit from Illumina (NEB) according to the manufacturer’s protocol. RNA sequencing was conducted by Beijing Novogene Bioinformatics Technology using Illumina NovaSeq 6000 sequencer (Illumina). To allow the comparison of gene expression profiles, values for each transcript were normalized and calculated as fragments per kilobase per million mapped reads (FPKM). Differentially expressed genes (DEGs) were calculated using the DEGseq package of R software [15]. Gene Ontology (GO), Disease Ontology (DO), and Kyoto Encyclopedia of Genes and Genomes (KEGG) pathway analysis were conducted to identify DEGs at the biologically functional level. The Database for Annotation, Visualization, and Integrated Discovery (DAVID) was used to integrate functional genomic annotations. *p* < 0.05 was considered to indicate a statistically significant difference.

### Reverse transcription quantitative real-time PCR (RT-qPCR)

Total RNA was extracted with the RNeasy Plus Mini Kit (QIAGEN) according to the manufacturer’s instructions. RNA (1 μg) was reverse-transcribed into first-strand cDNA with the iScript Reverse Transcription Kit (Bio-Rad Laboratories). Each 20-μL qPCR reaction contained 1X SYBR Green PCR Master Mix (Applied Biosystems), 60 ng of cDNA, and 250 nM of each specific primer. The following primers were used: KISS1, 5′-CAC TTT GGG GAG CCA TTA GA-3′ (sense) and 5′-CAG TAG CAG CTG GCT TCC TC-3′ (antisense); EGFR, 5′-GGT GCA GGA GAG GAG AAC TGC-3′ (sense) and 5′-GGT GGC ACC AAA GCT GTA TT-3′ (antisense); ID3, 5′-CGC GTC ATC GAC TAC ATT CT-3′ (sense) and 5′-GAG CTC GGC TGT CTG GAT-3′ (antisense); and GAPDH, 5′-GAG TCA ACG GAT TTG GTC GT-3′ (sense) and 5′-GAC AAG CTT CCC GTT CTC AG-3′ (antisense). qPCR was performed on an Applied Biosystems QuantStudio 12K Flex system equipped with 96-well optical reaction plates. The specificity of each assay was validated by melting curve analysis and agarose gel electrophoresis of the PCR products. All of the RT-qPCR experiments were run in triplicate, and a mean value was used to determine the mRNA levels. Water and mRNA without RT were used as negative controls. Relative quantification of the mRNA levels was performed using the comparative Ct method with GAPDH as the reference gene and using the formula 2^−∆∆Ct^.

### Western blot

Cells were lysed in cell lysis buffer (Cell Signaling Technology) supplemented with a protease inhibitor cocktail (Sigma). The protein concentration was analyzed by the BCA protein assay kit (Pierce, Thermo Scientific). Equal amounts (50 µg) of protein were separated by SDS polyacrylamide gel electrophoresis and transferred onto polyvinylidene difluoride (PVDF) membrane. After 1 h of blocking with 5% non-fat dry milk in Tris-buffered saline (TBS), the membranes were incubated overnight at 4 °C with a specific primary antibody diluted in 5% non-fat milk/TBS. The sources and dilutions for antibodies were: KISS1 (1:1000, abcam, #ab226786), ID3 (1:1000, Cell Signaling Technology, #9837), phospho-EGFR^Tyr1068^ (1:1000, Cell Signaling Technology, #3777), phospho-EGFR^Tyr1173^ (1:1000, Cell Signaling Technology, #4407), EGFR (1:2000, Cell Signaling Technology, #4267), phospho-HER2^Tyr1221/1222^ (1:1000, Cell Signaling Technology, #2243), HER2 (1:1000, Cell Signaling Technology, #2165), phospho-ERK1/2 (1:1000, Cell Signaling Technology, #9106), ERK1/2 (1:2000, Cell Signaling Technology, #9102), phospho-AKT (1:1000, Cell Signaling Technology, #9271), AKT (1:2000, Cell Signaling Technology, #9272), and α-tubulin (1:5000, Santa Cruz Biotechnology, #sc-23948). Following primary antibody incubation, the membrane was incubated with the appropriate HRP-conjugated secondary antibody. The immunoreactive band was detected using an enhanced chemiluminescent substrate (Bio-Rad Laboratories) and imaged with a ChemiDoc MP Imager (Bio-Rad Laboratories). Band intensities were quantified using the Scion Image software.

### Small interfering RNA (siRNA) transfection and overexpression

To knockdown endogenous EGFR, HTR-8/SVneo cells were transfected with 50 nM ON-TARGETplus SMARTpool siRNA targeting human EGFR (Dharmacon) using Lipofectamine RNAiMAX (Invitrogen). The same concentration of ON-TARGETplus siCONTROL NON-TARGETING pool siRNA (Dharmacon) was used as the transfection control. To overexpress ID3 or KISS1, cells were transfected with 1 µg empty pCMV vector or vector encoding a full-length of human ID3 or KISS1 (GeneChem, Shanghai) using Lipofectamine 3000 (Invitrogen).

### ELISA assay

KISS1 protein levels in culture media and human serum samples were measured using an enzyme-linked immunosorbent assay (ELISA). The Human KISS1 ELISA Kit (Elabscience, #E-EL-H6099) was used following the manufacturer’s protocol. Both interassay CV and intraassay CV for KISS1 ELISA were < 10%. The analytical sensitivity of KISS1 ELISA was 18.75 pg/mL. The Human EGF ELISA Kit (Elabscience, #E-EL-H0059) was used to measure EGF protein levels in human serum samples. Both interassay CV and intraassay CV for EGF ELISA were < 10%. The analytical sensitivity of EGF ELISA was 2.35 pg/mL.

### Invasion assay

Transwell cell culture inserts (8 µm pore size, 24 wells, BD Biosciences) were coated with 1 mg/mL growth factor-reduced Matrigel (BD Biosciences). Cells (1 × 10^5^ cells/insert) in DMEM/F-12 medium supplemented with 0.1% FBS were incubated for 48 h against a gradient of 10% FBS. Non-invasive cells were removed with a cotton swab from the upper side of the membrane. Cells that penetrated the membrane were fixed with cold methanol, stained with crystal violet (0.5%, Sigma) for 30 min, and subsequently washed thoroughly with tap water. Each experiment was performed with triplicate inserts. In each insert, five microscopic fields were photographed under an optical microscope, and the cell number was counted manually.

### Immunohistochemistry

The study received institutional approval (#2020-KY-164) and was carried out in accordance with the guidelines from the Zhengzhou University Research Ethics Board. Paraffin-embedded sections (5 μm) obtained from 3 control and 3 PE patients were deparaffinized and rehydrated. Antigen retrieval was conducted by boiling sections in sodium citrate buffer (pH 6.0) for 8 min. Endogenous peroxidase activity was blocked by incubating sections of 3% hydrogen peroxide solution at room temperature for 10 min. After 1 h of blocking with 3% bovine serum albumin in phosphate-buffered saline (PBS), sections were incubated with specific primary antibody overnight at 4 °C. Following primary antibody incubation (KISS1, 1:50, Santa Cruz Biotechnology #sc-101246; ID3, 1:50, OriGene #CF500714), the sections were incubated with HRP-conjugated secondary antibody. Sections were developed using the Peroxidase/DAB Dako REAL EnVision Detection System (Dako) and counterstained with hematoxylin. Negative control in the absence of primary antibody was performed in parallel.

### Statistical analysis

The results are presented as the mean ± SEM or mean ± SD. All statistical analyses were analyzed by PRISM software. For experiments involving only two groups, data were analyzed by *t* test. Multiple comparisons were analyzed using one-way ANOVA followed by Tukey’s multiple comparison test. A significant difference was defined as *p* < 0.05.

## Results

### RNA-seq identifies KISS1 as an EGF target gene in human trophoblast cells

**Fig. 1:**
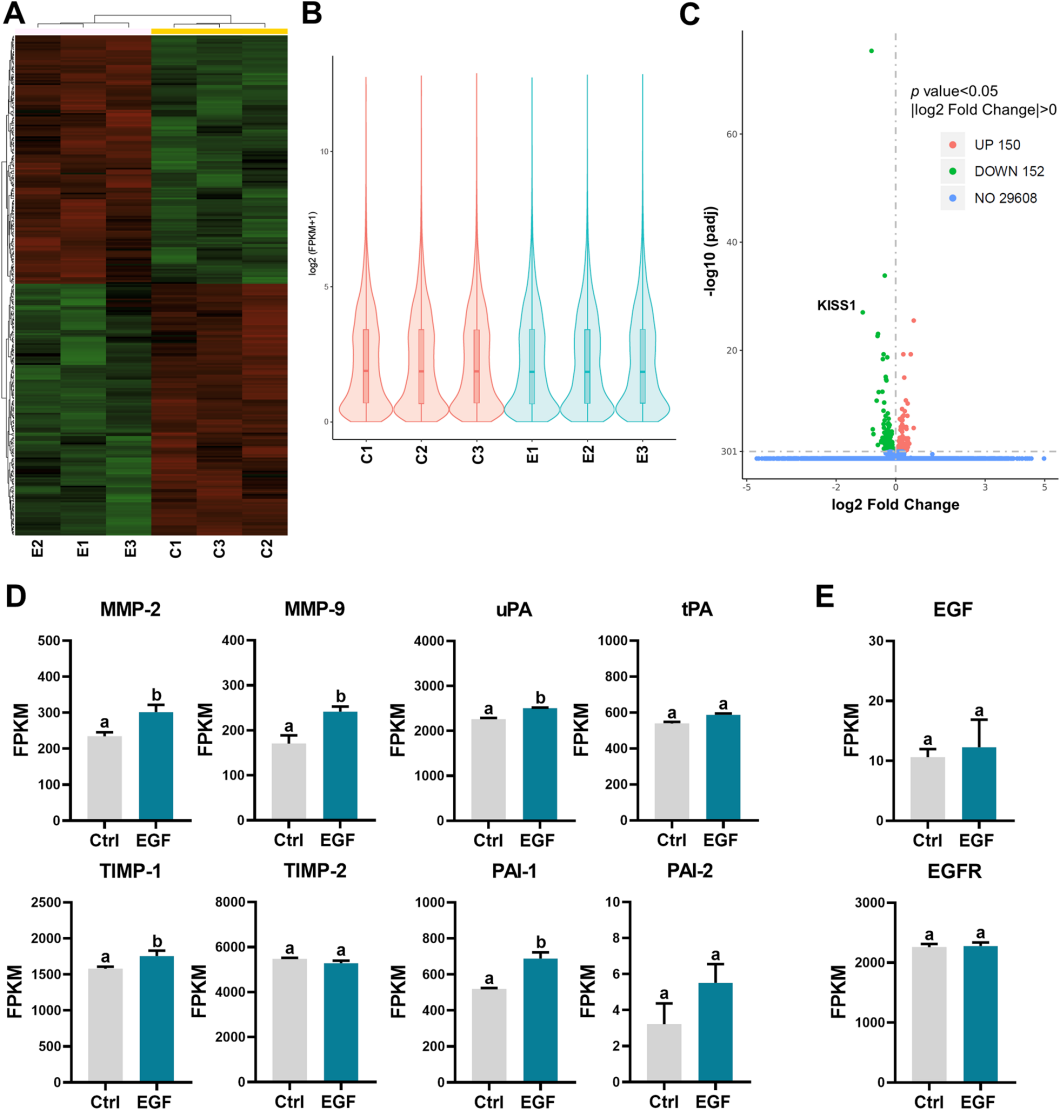
Transcriptome analysis of the effect of EGF on HTR-8/SVneo cells. **a** Cluster analysis of all the differentially expressed genes (DEGs) obtained from RNA-seq. **b** Violin plot of the expression patterns. **c** Volcano plot of RNA-seq data from control and EGF-treated cells. **d**, **e** Specific gene expression levels from RNA-seq data (FPKM, fragments per kilobase of exon per million reads) in control and EGF-treated cells

To explore the downstream target genes of EGF, RNA sequencing (RNA-seq) was performed on a human trophoblast cell line, HTR-8/SVneo, in triplicate after 24 h of 100 ng/mL EGF treatment. The result for the cluster analysis of transcriptome is presented in Fig. 1a. Gene expressions for control and EGF treatment groups are depicted in violin plots (Fig. 1b). RNA-seq results showed that 302 genes were significantly affected by EGF treatment, of which 150 were upregulated and 152 were downregulated (Fig. 1c). The detailed information for differentially expressed genes (DEGs) is presented in Additional file 1: Table S1. Gene Ontology (GO), Disease Ontology (DO), and Kyoto Encyclopedia of Genes and Genomes (KEGG) pathway analysis results are presented in Additional file 2 and 3: Figures S1 and S2. It is interesting to note that DO analysis indicated that downregulated genes were significantly enriched in the PE. Importantly, consistent with previous studies [10–13], RNA-seq results showed that EGF treatment significantly upregulated the mRNA levels of MMP-2, MMP-9, TIMP-1, uPA, and PAI-1. The mRNA levels of TIPM-2, tPA, and PAI-2 were not affected by EGF (Fig. 1d). In addition, treatment with EGF for 24 h did not influence the expression of EGF and EGFR (Fig. 1e).

### EGF downregulates KISS1 through EGFR

**Fig. 2:**
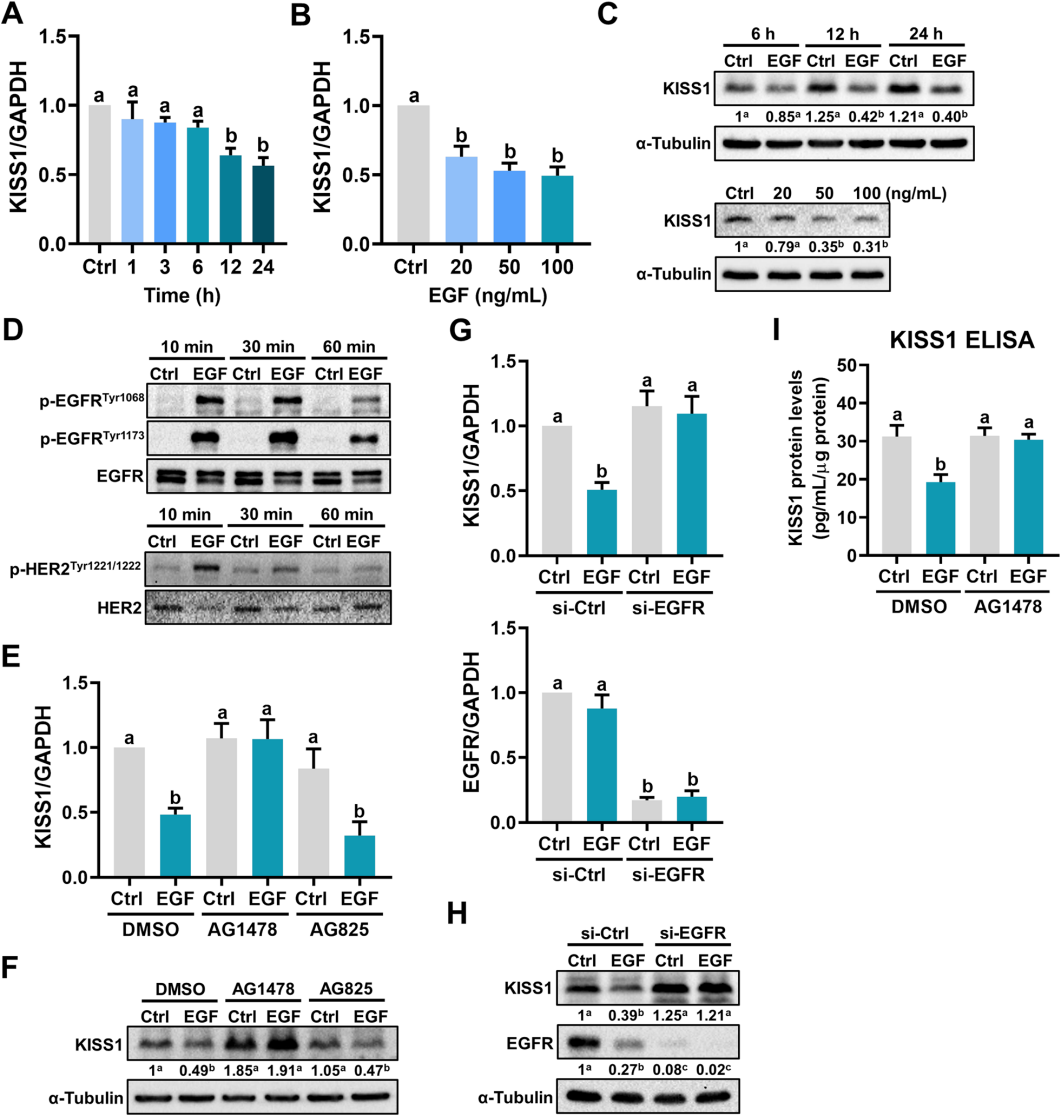
EGF downregulates KISS1 expression and secretion in human trophoblast cells. **a**–**c** HTR-8/SVneo cells were treated with 100 ng/mL EGF for different periods (**a**) or treated with different concentrations of EGF for 24 h (**b**). The KISS1 mRNA levels (**a**, **b**) and protein levels (**c**) were examined by RT-qPCR and western blot, respectively. **d** HTR-8/SVneo cells were treated with 50 ng/mL EGF for 10, 30, and 60 min. EGFR phosphorylation levels at Tyr1068 and Tyr1173, HER2 phosphorylation levels at Tyr1221/1222 were examined by western blot. **e** and **f** HTR-8/SVneo cells were pretreated with vehicle control (DMSO), 5 µM AG1478, or 5 µM AG825 for 1 h, and then treated with 50 ng/mL EGF for 24 h. The KISS1 mRNA levels (**e**) and protein levels (**f**) were examined by RT-qPCR and western blot, respectively. **g**, **h**, HTR-8/Svneo cells were transfected with 50 nM control siRNA (si-Ctrl) or EGFR siRNA (si-EGFR) for 48 h, and then treated with 50 ng/mL EGF for 24 h. The mRNA (**g**) and protein (**h**) levels of KISS1 and EGFR were examined by RT-qPCR and western blot, respectively. **i**, HTR-8/SVneo cells were pretreated with vehicle control (DMSO) or 5 µM AG1478 for 1 h, and then treated with 50 ng/mL EGF every 24 h for 48 h. The protein levels of KISS1 in culture media were examined by ELISA. Numbers under the western blots represent the densitometry quantifications. The results are expressed as the mean ± SEM of at least three independent experiments. Values without a common letter are significantly different (*p* < 0.05)

KISS1 was first identified as a human melanoma metastasis suppressor gene in 1996 [16]. KISS1 gene encodes kisspeptin, also known as metastin, which belongs to the family of RF-amide peptides and suppresses tumor metastasis by binding to a G protein-coupled receptor, GPR54 [17]. RNA-seq results showed that although KISS1 mRNA levels were downregulated by EGF treatment, the mRNA levels of GPR54 were not affected by EGF (Additional file 4: Figure S3). To further confirm the RNA-seq results, HTR-8/SVneo cells were treated with 100 ng/mL for different periods and the expression of KISS1 was examined. As shown in Fig. 2a, EGF treatment resulted in a downregulation of KISS1 mRNA levels in a time-dependent manner. Treatment with 20, 50, or 100 ng/mL EGF showed a comparable inhibitory effect on KISS1 mRNA levels (Fig. 2b). In addition, western blot results confirmed the inhibitory effect of EGF on the protein levels of KISS1 (Fig. 2c). Therefore, 50 ng/mL EGF was used for the following experiments. EGF binds specifically to EGFR. Besides, although no direct identified ligand, HER2 can be activated via dimerization with EGF-activated EGFR to enhance EGFR signaling [18]. Treatment with EGF activated both EGFR and HER2 in HTR-8/SVneo cells (Fig. 2d). To examine the involvement of EGFR and HER2 in EGF-downregulated KISS1 expression, EGFR inhibitor AG1478, and HER2 inhibitor AG825 were used to block their function, respectively. The EGF-activated EGFR or HER2 was blocked by pretreatment with EGFR inhibitor AG1478 or HER2 inhibitor AG825, respectively (Additional file 5: Figure S4). Inhibition of EGFR blocked the EGF-induced downregulation of KISS1 expression. Interestingly, activation of HER2 was not required for the EGF-induced downregulation of KISS1 expression (Fig. 2e, f). To further confirm the requirement of EGFR for the inhibitory effect of EGF on KISS1 expression, a siRNA-mediated knockdown approach was used to block the function of EGFR. As shown in Fig. 2g, h, knockdown of EGFR blocked the EGF-induced downregulation of KISS1 mRNA and protein levels. Upon EGF binding to the EGFR, the clustering and internalization of the ligand and receptor complex are induced, with the subsequent lysosomal degradation of both the ligand and receptor [19]. As shown in Fig. 2h, our results supported this process and showed that EGFR was down-regulated in response to EGF treatment. Importantly, ELISA results showed that the KISS1 protein was secreted by HTR-8/SVneo cells. Treatment with EGF reduced the KISS1 protein secretion and this inhibitory effect was blocked by inhibition of EGFR (Fig. 2i).

### PI3K/AKT signaling pathway is involved in EGF-induced downregulation of KISS1

**Fig. 3:**
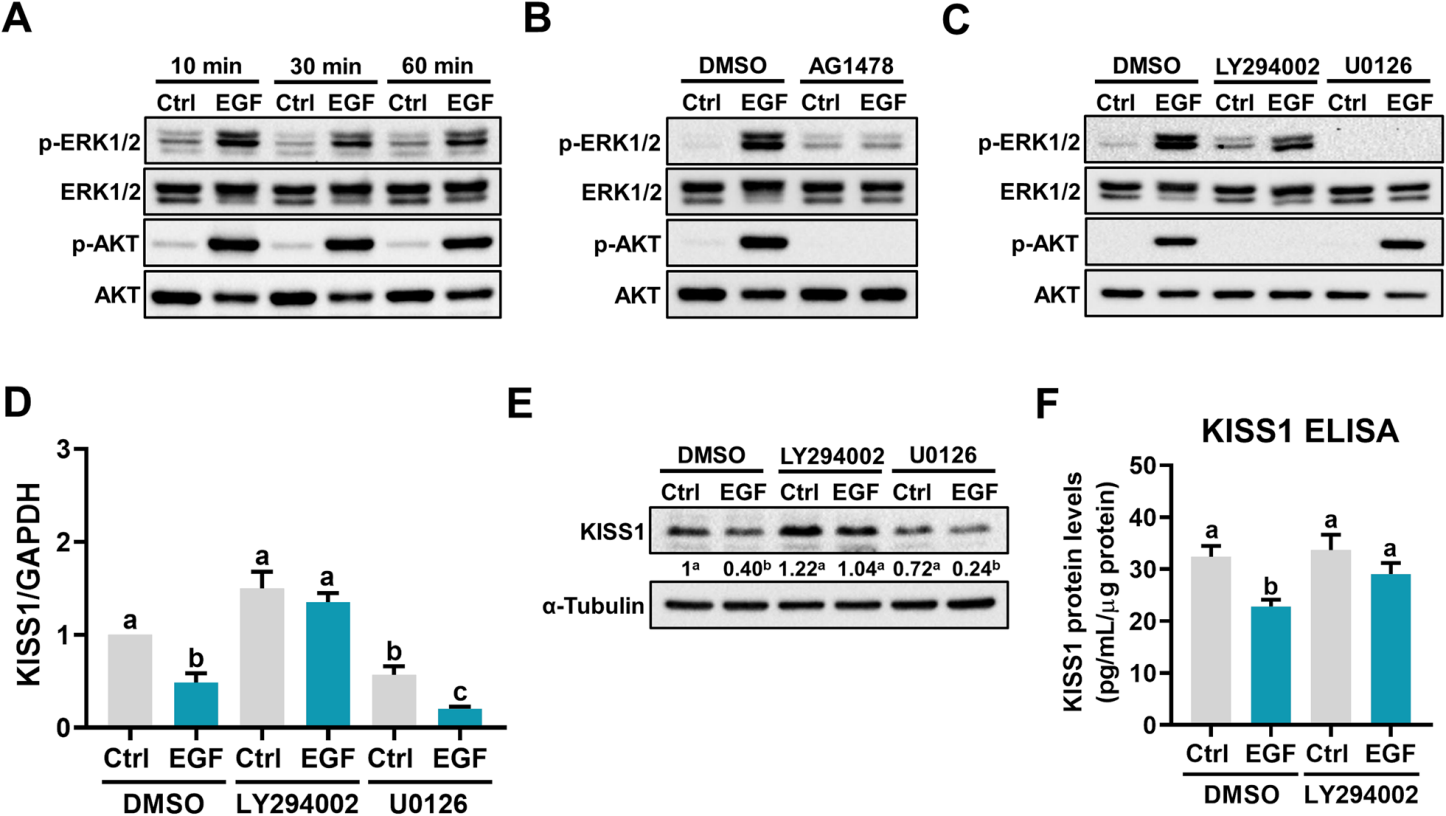
Activation of PI3K/AKT signaling pathway is involved in EGF-suppressed KISS1 expression and secretion. **a**–**c**, HTR-8/SVneo cells were treated with 50 ng/mL EGF for 10, 30, and 60 min (**a**). HTR-8/SVneo cells were pretreated with vehicle control (DMSO) or 5 µM AG1478 for 1 h, and then treated with 50 ng/mL EGF for 10 min (**b**). HTR-8/SVneo cells were pretreated with vehicle control (DMSO), 5 µM LY294002, or 5 µM U0126 for 1 h, and then treated with 50 ng/mL EGF for 10 min (**c**). The levels of phosphorylated and total forms of ERK1/2 and AKT were determined by western blot. **d**, **e** HTR-8/SVneo cells were pretreated with vehicle control (DMSO), 5 µM LY294002, or 5 µM U0126 for 1 h, and then treated with 50 ng/mL EGF for 24 h. The KISS1 mRNA levels (**d**) and protein levels (**e**) were examined by RT-qPCR and western blot, respectively. **f** HTR-8/SVneo cells were pretreated with vehicle control (DMSO) or 5 µM LY294002 for 1 h, and then treated with 50 ng/mL EGF every 24 h for 48 h. The protein levels of KISS1 in culture media were examined by ELISA. Numbers under the western blots represent the densitometry quantifications. The results are expressed as the mean ± SEM of at least three independent experiments. Values without a common letter are significantly different (*p* < 0.05)

To explore the intracellular signaling pathways that mediate the EGF-induced we focused on the ERK1/2 and PI3K/AKT because they are the most well-characterized EGFR downstream signaling pathways [20]. Treatment of HTR-8/SVneo cells with EGF activated both ERK1/2 and PI3K/AKT signaling pathways (Fig. 3a). The stimulatory effects of EGF on ERK1/2 and PI3K/AKT activation were blocked by inhibition of EGFR (Fig. 3b). To determine the involvement of these two signaling pathways in EGF-induced downregulation of KISS1 expression, the MEK inhibitor U0126 and PI3K inhibitor LY294002 were used to block the activation of ERK1/2 and AKT, respectively (Fig. 3c). As shown in Fig. 3d, e, inhibition of the AKT signaling attenuated the inhibitory effect of EGF on KISS1 mRNA and protein levels. However, inhibition of the ERK1/2 signaling pathway did not affect the EGF-induced downregulation of KISS1 expression. Moreover, inhibition of the AKT signaling pathway attenuated the EGF-reduced KISS1 protein secretion (Fig. 3f).

### EGF downregulates KISS1 by inhibiting ID3 expression

**Fig. 4:**
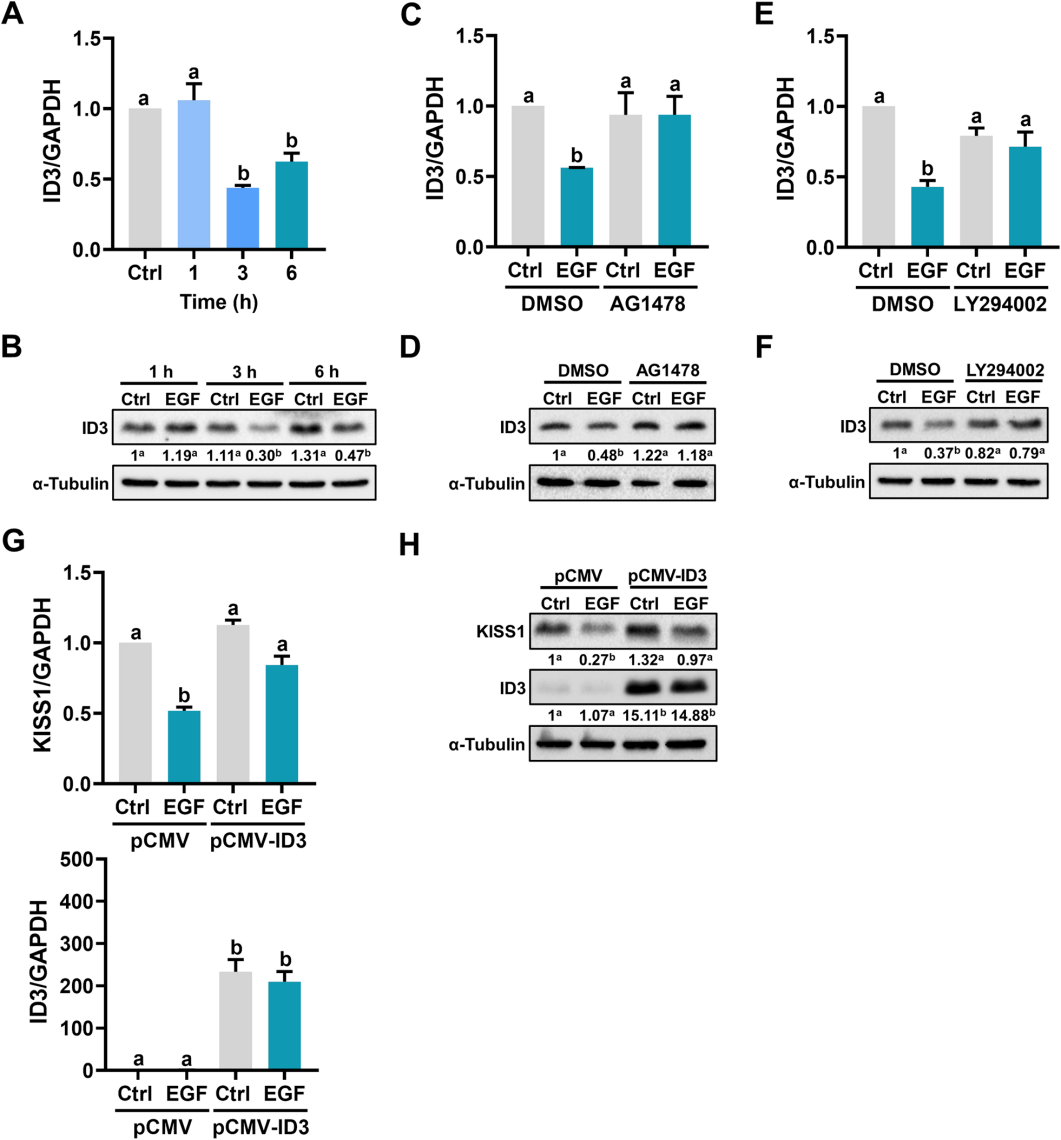
Downregulation of ID3 mediates the EGF-suppressed KISS1 expression. **a**, **b** HTR-8/SVneo cells were treated with 50 ng/mL EGF for 1, 3, and 6 h. The ID3 mRNA levels (**a**) and protein levels (**b**) were examined by RT-qPCR and western blot, respectively. **c**, **d** HTR-8/SVneo cells were pretreated with vehicle control (DMSO) or 5 µM AG1478 for 1 h, and then treated with 50 ng/mL EGF for 3 h. The ID3 mRNA levels (**c**) and protein levels (**d**) were examined by RT-qPCR and western blot, respectively. **e**, **f** HTR-8/SVneo cells were pretreated with vehicle control (DMSO) or 5 µM LY294002 for 1 h, and then treated with 50 ng/mL EGF for 3 h. The ID3 mRNA levels (**e**) and protein levels (**f**) were examined by RT-qPCR and western blot, respectively. **g**, **h** HTR-8/SVneo cells were transfected with 1 µg control vector (pCMV) or vector containing human ID3 cDNA (pCMV-ID3) for 48 h. After transfections, cells were treated with 50 ng/mL EGF for 24 h. The mRNA (**g**) and protein (**h**) levels of KISS1 and ID3 were examined by RT-qPCR and western blot, respectively. Numbers under the western blots represent the densitometry quantifications. The results are expressed as the mean ± SEM of at least three independent experiments. Values without a common letter are significantly different (*p* < 0.05)

The inhibitor of DNA-binding (ID) proteins are transcriptional regulators. Because lacking the DNA-binding domain, ID proteins can heterodimerize with bHLH transcription factors to inhibit their DNA binding activity [21]. It is known that ID3 participates in several EGFR-mediated cellular functions [22]. Treatment of HTR-8/SVneo cells for 3 h downregulated ID3 mRNA and protein levels (Fig. 4a, b). The inhibitory effects of EGF on ID3 mRNA and protein levels were blocked by the inhibition of EGFR (Fig. 4c, d). In addition, inhibition of the PI3K/AKT signaling pathway also blocked the EGF-induced downregulation of ID3 expression (Fig. 4e, f). To examine the role of ID3 in EGF-induced KISS1 downregulation, ID3 was overexpressed in HTR-8/SVneo cells and KISS1 expression was examined. RT-qPCR and western blot results showed that cells transfected with ID3 expressed more ID3 while cells transfected with empty vector did not. However, overexpression of ID3 did not affect the basal levels of KISS1 expression. Importantly, EGF-reduced KISS1 mRNA and protein levels were attenuated in cells overexpressing ID3 (Fig. 4g, h).

### EGF stimulates trophoblast cell invasion by inhibiting KISS1 expression

**Fig. 5:**
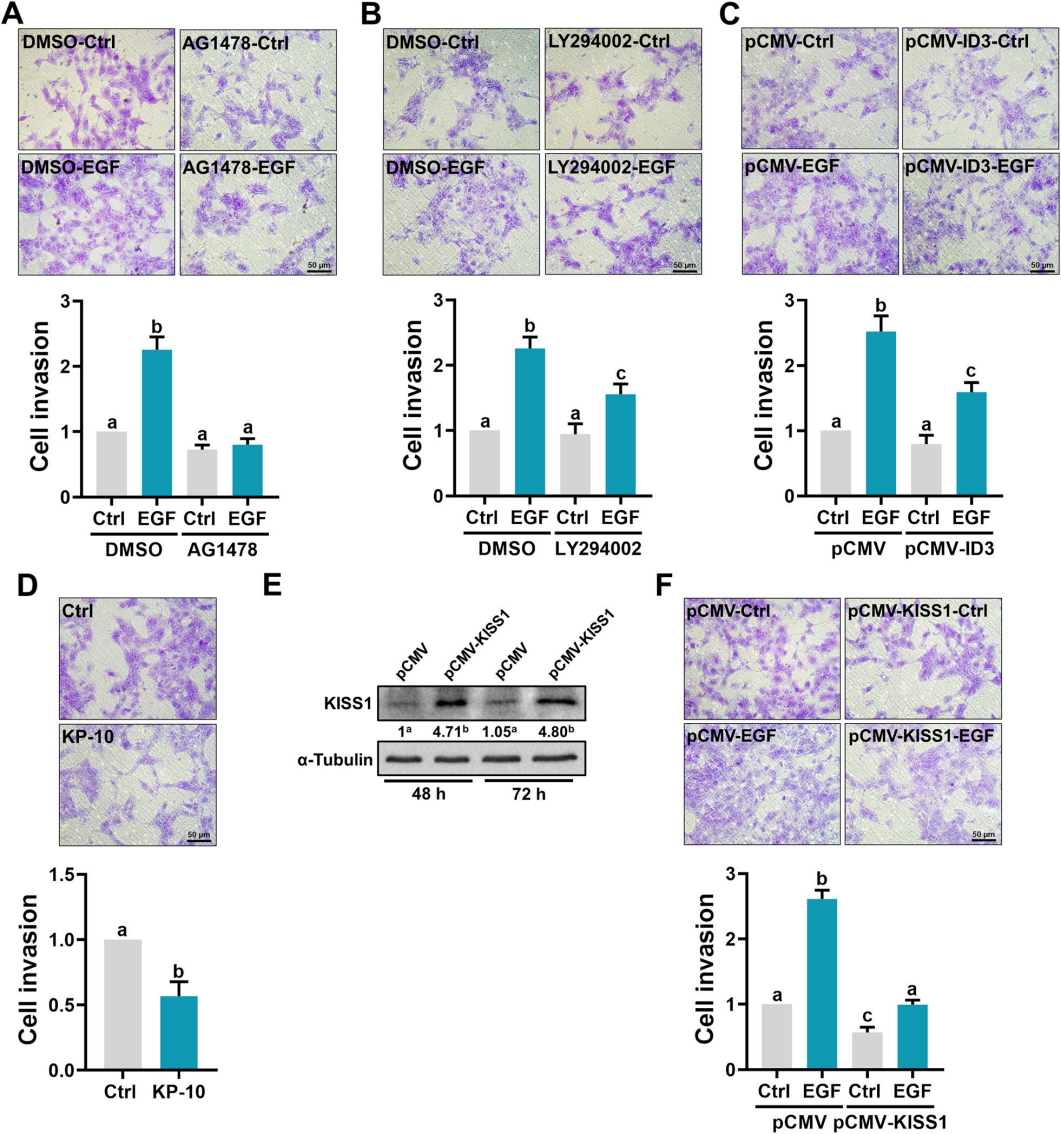
EGF promotes trophoblast cell invasion by downregulating KISS1 expression. **a**, **b**, HTR-8/SVneo cells were pretreated with vehicle control (DMSO), 5 µM AG1478 (**a**), or 5 µM LY294002 (**b**) for 1 h, and then treated with 50 ng/mL EGF. After treatments, cells were seeded onto Matrigel-coated transwell inserts. After 48 h of incubation, noninvading cells were wiped from the upper side of the filter, and the nuclei of the invading cells were stained with crystal violet. **c** HTR-8/SVneo cells were transfected with 1 µg control vector (pCMV) or vector containing human ID3 cDNA (pCMV-ID3) for 48 h. After transfections, cells were treated with 50 ng/mL EGF and the levels of cell invasiveness were examined by transwell invasion assay. **d** HTR-8/SVneo cells were treated with 1 µM kisspeptin-10 (KP-10) and the levels of cell invasiveness were examined by transwell invasion assay. **e** HTR-8/SVneo cells were transfected with 1 µg control vector (pCMV) or vector containing human KISS1 cDNA (pCMV-KISS1) for 48 and 72 h. The protein levels of KISS1 were examined by western blot. Numbers under the western blots represent the densitometry quantifications. **f** HTR-8/SVneo cells were transfected with 1 µg control vector (pCMV) or vector containing human KISS1 cDNA (pCMV-KISS1) for 48 h. After transfections, cells were treated with 50 ng/mL EGF and the levels of cell invasiveness were examined by transwell invasion assay. The invasion assay results are expressed as the mean ± SEM of at least three independent experiments. Values without a common letter are significantly different (*p* < 0.05)

Consistent with previous studies, EGF treatment stimulated the invasiveness of HTR-8/SVneo cells. The pro-invasive effect of EGF on HTR-8/SVneo cells was blocked by inhibition of EGFR (Fig. 5a). In addition, inhibition of the PI3K/AKT signaling pathway attenuated the EGF-stimulated cell invasion (Fig. 5b). Given the role of ID3 in the EGF-reduced KISS1 expression, we examined the cell invasiveness after ID3 overexpression. As shown in Fig. 5c, overexpression of ID3 did not affect the basal levels of cell invasiveness, but it attenuated the EGF-stimulated cell invasiveness (Fig. 5c). To examine the role of KISS1 in trophoblast cell invasion, the invasiveness of HTR-8/SVneo cells was examined after treatment with the human recombinant kisspeptin-10 (KP-10) which has been shown to inhibit primary human trophoblast cell invasion [23]. As shown in Fig. 5d, treatment with KP-10 reduced the invasiveness of HTR-8/SVneo cells. To further examine the involvement of KISS1 in EGF-stimulated cell invasiveness, KISS1 was overexpressed in HTR-8/SVneo cells and the cell invasiveness was examined. As shown in Fig. 5e, KISS1 overexpression significantly increased the KISS1 protein levels after 48 and 72 h of transfection. Importantly, overexpression of KISS1 not only reduced basal cell invasiveness but also attenuated EGF-stimulated cell invasiveness in HTR-8/SVneo cells (Fig. 5f).

### KISS1 expression is upregulated in the serum and placenta of PE patients

**Fig. 6:**
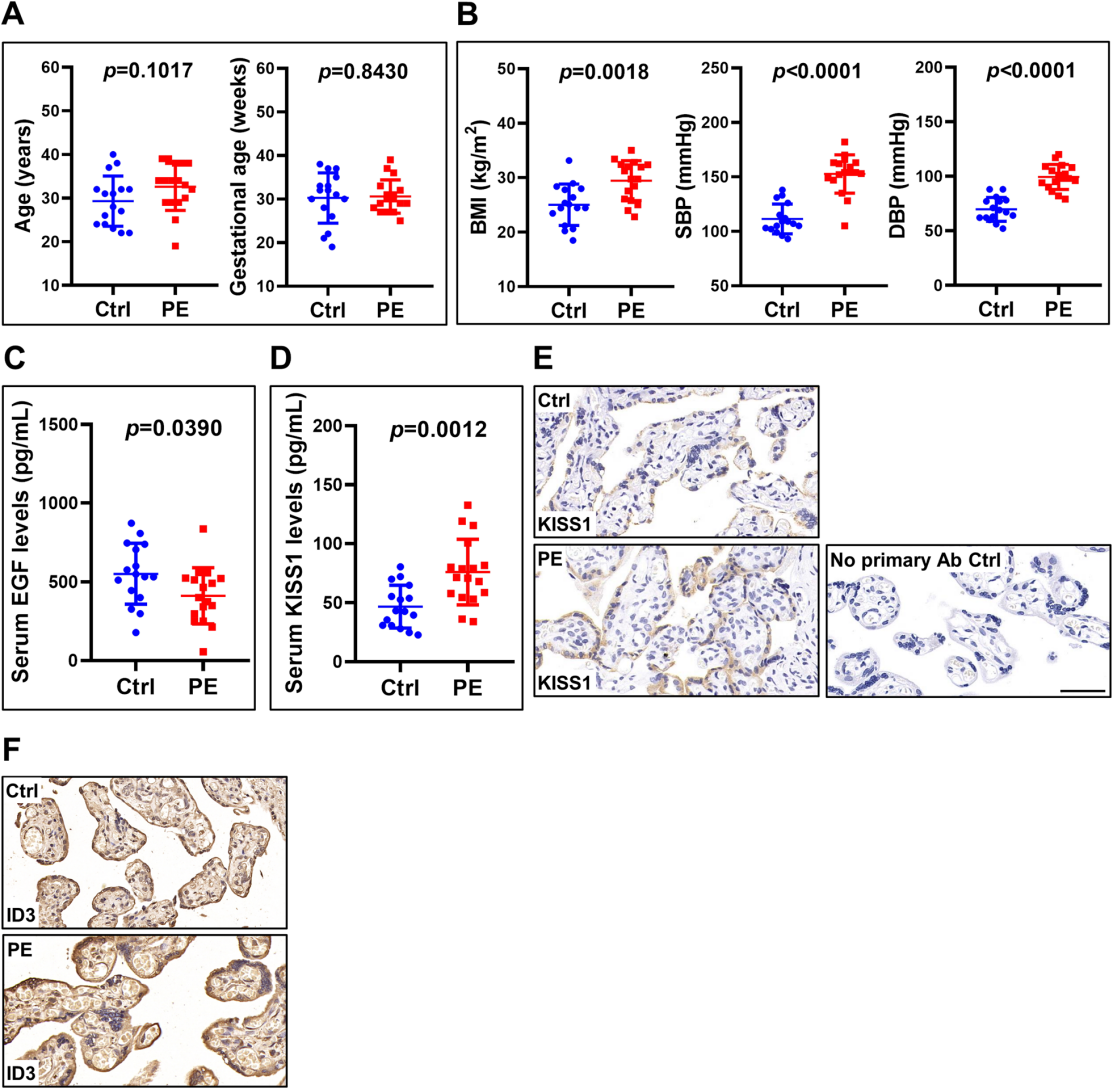
KISS1 and ID3 expression levels are upregulated in the placenta of PE patients. **a**, **b**, Serum samples were collected from 17 PE patients and 16 normal pregnant women of similar age and gestational age. The age and gestational age of the included patients are presented (**a**). body mass index (BMI), systolic blood pressure (SBP), and diastolic blood pressure (DBP) were measured (**b**). **c** Serum levels of EGF were measured by ELISA. **d** Serum levels of KISS1 were measured by ELISA. **e**, **f** Representative images of immunohistochemical staining for KISS1 (**e**) and ID3 (**f**) in the control and PE placenta. Original magnification: ×200. The scale bar represents 50 μm. The clinical results are expressed as the mean ± SD

Given the anti-invasive role of KISS1 in human trophoblast cells, we next compared serum KISS1 levels in patients with and without PE. KISS1 serum levels were measured in 17 PE patients and 16 normal pregnant women of similar age and gestational age (Fig. 6a). Consistent with previous reports, Body mass index (BMI), systolic blood pressure (SBP), and diastolic blood pressure (DBP) were significantly upregulated in PE patients compared with normal controls (Fig. 6b) [6, 24]. In addition, similar to the previous study, serum EGF levels were reduced in PE patients when compared to those of control patients [8] (Fig. 6c). Interestingly, ELISA results showed that KISS1 serum levels were significantly upregulated in PE patients (Fig. 6d). Immunohistochemical staining showed a similar result that KISS1 expression was upregulated in the placenta of PE patients (Fig. 6e). In addition, the expression of ID3 was upregulated in the placenta of PE patients (Fig. 6f). Taken together, these results demonstrate that reduced EGF levels result in the upregulation of KISS1 and ID3 expressions in PE patients and that could lead to the poor trophoblast cell invasion which is commonly observed in PE.

## Discussion

The pro-invasive role of EGF in human trophoblast cells has been reported. In addition, aberrant expressions of EGF and EGFR are associated with trophoblast cell invasion-related placental diseases. However, the underlying mechanism that mediates the pro-invasive effect of EGF on human trophoblast cells remains very limited. In the present study, using RNA-seq we identified that KISS1 was the downstream target gene of EGF/EGFR in human trophoblast cells. KISS1 and its receptor are abundantly expressed in the human placenta [25]. KISS1 is a placenta-derived hormone. The plasma levels of KISS1 are dramatically increased during pregnancy and return to basal levels after giving birth. The human KISS1 gene initially encodes an unstable and biologically inactive 145 amino acid prepropeptide which can be further cleaved into 4 different lengths of biologically active peptides and named by their number of amino acids: kisspeptin-54 (KP-54), KP-14, KP-13, and KP-10 [26]. Different forms of KP have the same affinity and binding efficacy onto their receptor, GPR54 [27]. It has been shown that KP-10 treatment inhibits cell migration and invasion in primary human trophoblast cells and HTR-8/SVneo cells [23, 28, 29]. Interestingly, the reduction of cell invasiveness is attributed to the KP-10-downregulated MMPs expression [23, 29]. Upregulation of MMPs expression is involved in EGF-stimulated human trophoblast cell invasion [10, 11]. Collectively, these studies together with our results indicate that KISS1 is the downstream target of EGF/EGFR in human trophoblast cells. In addition, EGF can stimulate human trophoblast cell invasion by downregulating KISS1 expression and inhibiting KISS1-suppressed MMPs expression.

PE is characterized by insufficient trophoblast invasion. In the present study, we showed that the serum levels of KISS1 were upregulated in PE patients when compared to normal controls. However, our results conflicted with previous studies showing that serum or plasma KISS1 levels are downregulated in PE patients [30, 31]. Although we do not know the exact factor that causes these controversial results, different methods for KISS1 measurement could lead to this observation. In addition, previous studies have shown that the plasma levels of KISS1 may not necessarily reflect the expression levels of KISS1 in the placenta [32]. Nevertheless, consistent with a recent study [33], our immunostaining results showed that the placental protein expression of KISS1 was upregulated in PE patients. Our ELISA results indicated that KISS1 was able to be secreted by human trophoblast cells. Taken together, these results suggest that KISS1 may act as a local factor that regulates trophoblast cell invasion in an autocrine/paracrine fashion. To this end, it will be interesting to compare the expression level of KISS1 in the amniotic fluid samples of control and PE patients.

ID3 is expressed in the human placenta [34]. In the present study, we demonstrated that the expression of ID3 was downregulated by EGF and that was required for the EGF-suppressed KISS1 expression in human trophoblast cells. In human lung cancer cells, overexpression of ID3 inhibits cell proliferation, migration, invasion, and tumorigenesis, although the underlying mechanisms remain undetermined [35]. To the best of our knowledge, the function of ID3 protein in the regulation of trophoblast cell invasion is completely unknown. Our transwell matrigel invasion assay showed that overexpression of ID3 did not affect the basal cell invasiveness but attenuated the EGF-stimulated cell invasiveness in HTR-8/SVneo cells. The anti-invasive effect of ID3 on EGF-stimulated trophoblast cell invasion was mediated by regulating KISS1 expression. ID3 protein does not have the basic DNA binding domain but it can act as a regulator for gene expression by forming heterodimers with other bHLH transcription factors [36]. To date, the involvement of specific transcription factors in the regulation of KISS1 gene expression has not been well characterized. Previous studies have shown that two bHLH transcription factors, PTF1A and TCF21, can regulate KISS1 expression [37, 38]. Whether ID3 affects KISS1 expression by interacting with these bHLH transcription factors remains unclear and needs further investigation.

The HTR-8/SVneo cell line was generated using first-trimester extravillous trophoblast cells infected with simian virus 40 large T antigen [14]. Thus far, this cell line is the most commonly used experimental model for studying the biology of human trophoblast cells. Comparing to the primary culture of human trophoblast cells, using HTR-8/SVneo cells as an experimental model makes the experiments more technically feasible, particularly for those involving gene knockdowns or overexpression. Nevertheless, we are aware that the HTR-8/SVneo cell line can not fully represent the nature of normal human trophoblast cells and the results of the present study were derived mainly from the HTR-8/SVneo cells. Therefore, the findings reported by the present could be further examined in primary human trophoblast cells.

## Conclusions

In summary, using RNA-seq, we identify KISS1 as an EGF/EGFR target gene in human trophoblast cells. EGF treatment downregulates KISS1 expression and secretion by activating the EGFR-mediated PI3K/AKT signaling pathway. In addition, we show that ID3 protein is downregulated by EGF and that is required for EGF-suppressed KISS1 expression. EGF stimulates human trophoblast cell invasion by downregulating KISS1 expression. Furthermore, clinical results reveal that serum levels of EGF are downregulated while serum and placental levels of KISS1 are upregulated in PE patients. These results suggest that downregulation of EGF can lead to poor trophoblast cell invasion by increasing KISS1 expression which subsequently contributes to the pathogenesis of PE. Our study not only provides important insights into the regulation of KISS1 expression but also increases the understanding of the biological roles of EGF/EGFR in the human placenta.

## Supplementary Information

**Additional file 1:** **Table S1.** The detailed information for differentially expressed genes (DEGs).

**Additional file 2:** **Figure S1.** Gene Ontology (GO) and Disease Ontology (DO) analysis of DEGs.

**Additional file 3:** **Figure S2.** KEGG signaling pathway analysis of DEGs.

**Additional file 4:** **Figure S3.** RNA-seq results of KISS1 and GPR54 levels.

**Additional file 5:** **Figure S4.** The effect of AG1478 on EGFR activation and the effect of AG825 on HER2 activation. HTR-8/SVneo cells were pretreated with vehicle control (DMSO), 5 μM AG1478 (left panel), or 5 μM AG825 for 1 h, and then treated with 50 ng/mL EGF for 10 min. EGFR phosphorylation levels at Tyr1068 and Tyr1173, and HER2 phosphorylation levels at Tyr1221/1222 were examined by western blot.

## Data Availability

The data that support the findings of this study are available from the corresponding author upon reasonable request.

## Abbreviations

BMI: Body mass index
DAVID: Database for Annotation, Visualization, and Integrated Discovery
DBP: Diastolic blood pressure
DEGs: Differentially expressed genes
DO: Disease Ontology
ECM: Extracellular matrix
EGF: Epidermal growth factor
EGFR: Epidermal growth factor receptor
ELISA: Enzyme-linked immunosorbent assay
FPKM: Fragments per kilobase per million mapped reads
GO: Gene Ontology
ID: Inhibitor of DNA-binding protein
IUGR: Intrauterine growth restriction
KEGG: Kyoto Encyclopedia of Genes and Genomes
MMP: Metalloproteinase
PAI-1: Plasminogen activator inhibitor-1
PE: Preeclampsia
PVDF: Polyvinylidene difluoride
RNA-seq: RNA sequencing
SBP: Systolic blood pressure
TIMP-1: Tissue inhibitor of metalloproteinases-1
uPA: Urokinase plasminogen activator
