# Supplementary material for: EGF stimulates human trophoblast cell invasion by downregulating ID3-mediated KISS1 expression
Source: Cell Commun Signal. 2021 Oct 7;19:101. doi: 10.1186/s12964-021-00783-2 (PMC8499481; doi:10.1186/s12964-021-00783-2)
Supplement: Supplementary file 3 — Additional file 2: Figure S1. Gene Ontology (GO) and Disease Ontology (DO) analysis of DEGs. [file 12964_2021_783_MOESM3_ESM.pdf]

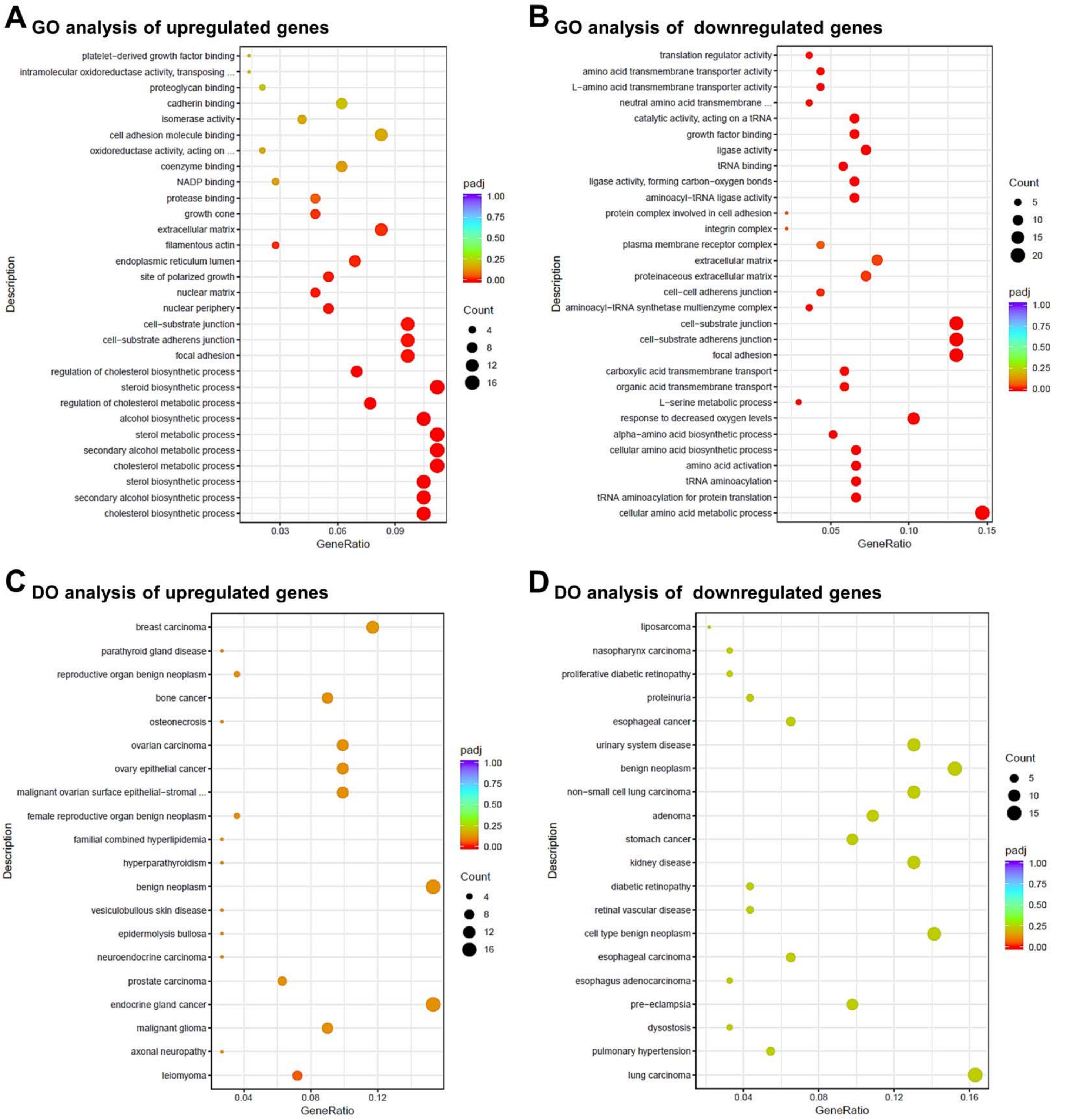

Additional file 2: Figure S1. Gene Ontology (GO) and Disease Ontology (DO) analysis of DEGs.
