# Supplementary material for: EGF stimulates human trophoblast cell invasion by downregulating ID3-mediated KISS1 expression
Source: Cell Commun Signal. 2021 Oct 7;19:101. doi: 10.1186/s12964-021-00783-2 (PMC8499481; doi:10.1186/s12964-021-00783-2)
Supplement: Supplementary file 4 — Additional file 3: Figure S2. KEGG signaling pathway analysis of DEGs. [file 12964_2021_783_MOESM4_ESM.pdf]

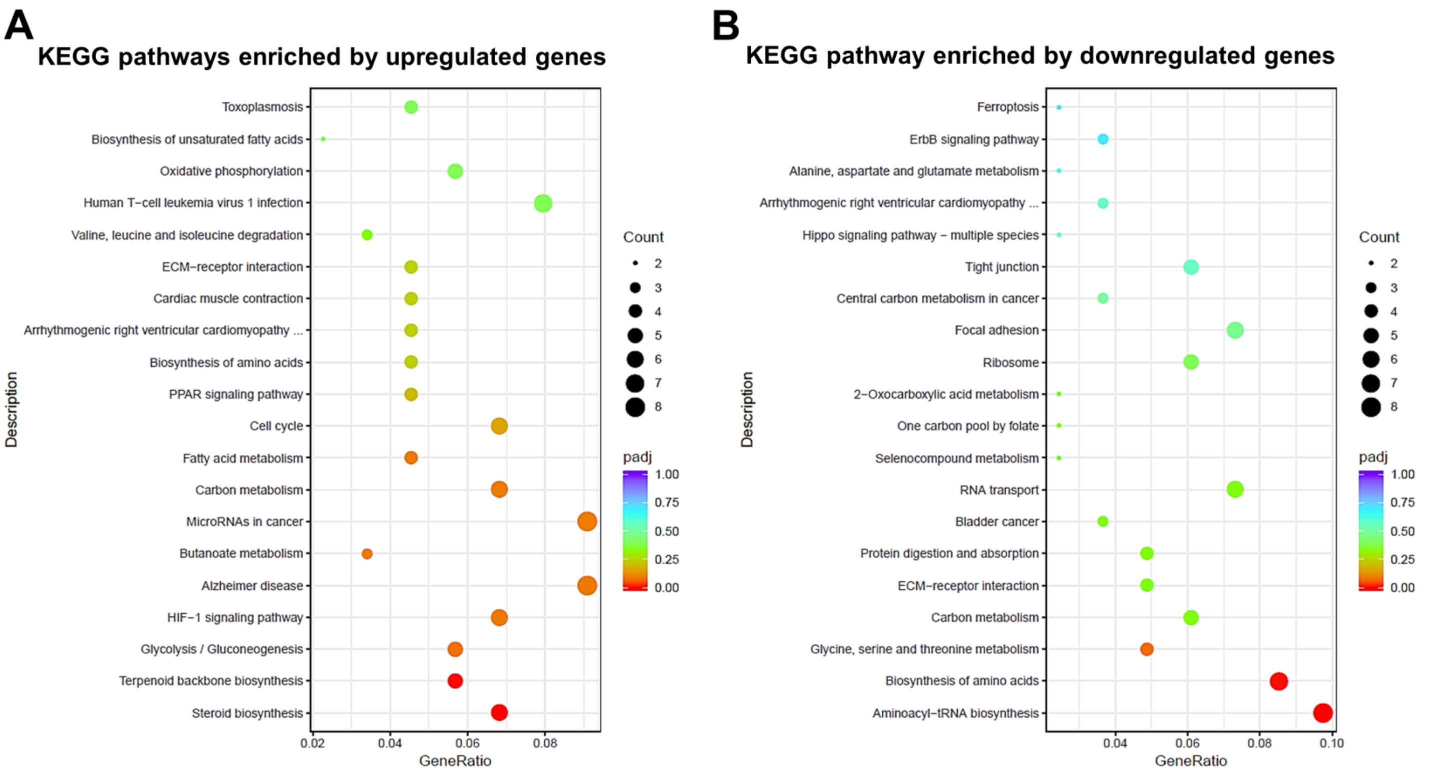

Additional file 3: Figure S2. KEGG signaling pathway analysis of DEGs.
