# Supplementary figures and images for: EGF stimulates human trophoblast cell invasion by downregulating ID3-mediated KISS1 expression
Source: Cell Commun Signal. 2021 Oct 7;19:101. doi: 10.1186/s12964-021-00783-2 (PMC8499481; doi:10.1186/s12964-021-00783-2)

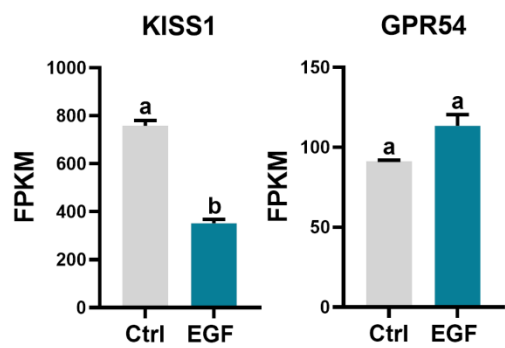

**Additional file 4: Figure S3.** RNA-seq results of KISS1 and GPR54 levels.

Supplement: Supplementary file 5 — Additional file 4: Figure S3. RNA-seq results of KISS1 and GPR54 levels. [file 12964_2021_783_MOESM5_ESM.pdf]
