# Supplementary material for: EGF stimulates human trophoblast cell invasion by downregulating ID3-mediated KISS1 expression
Source: Cell Commun Signal. 2021 Oct 7;19:101. doi: 10.1186/s12964-021-00783-2 (PMC8499481; doi:10.1186/s12964-021-00783-2)
Supplement: Supplementary file 6 — Additional file 5: Figure S4. The effect of AG1478 on EGFR activation and the effect of AG825 on HER2 activation. HTR-8/SVneo cells were pretreated with vehicle control (DMSO), 5 μM AG1478 (left panel), or 5 μM AG825 for 1 h, and then treated with 50 ng/mL EGF for 10 min. EGFR phosphorylation levels at Tyr1068 and Tyr1173, and HER2 phosphorylation levels at Tyr1221/1222 were examined by western blot. [file 12964_2021_783_MOESM6_ESM.pdf]

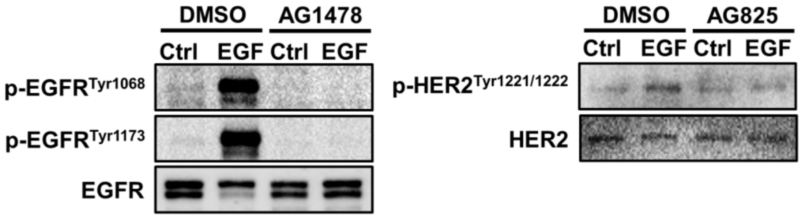

Additional file 5: Figure S4. The effect of AG1478 on EGFR activation and the effect of AG825 on HER2 activation. HTR-8/SVneo cells were pretreated with vehicle control (DMSO), 5 μM AG1478 (left panel), or 5 μM AG825 for 1 h, and then treated with 50 ng/mL EGF for 10 min. EGFR phosphorylation levels at Tyr1068 and Tyr1173, and HER2 phosphorylation levels at Tyr1221/1222 were examined by western blot.
